# Multifunctional coil technique for alignment-agnostic and Rx coil size-insensitive efficiency enhancement for wireless power transfer applications

**DOI:** 10.1038/s41598-023-50094-4

**Published:** 2023-12-21

**Authors:** Seoyeon Yoon, Taejun Lim, Yongshik Lee

**Affiliations:** Department of Electrical and Electronic Engineering, Yonsei University, Seoul, 03722 South Korea

**Keywords:** Electrical and electronic engineering, Engineering

## Abstract

This paper presents a multifunctional coil technique to enhance the transfer efficiency of an inductively-coupled wireless power transfer (WPT) system, regardless of the alignment condition and size ratio between the transmitter (Tx) and receiver (Rx) coils. The technique incorporates an auxiliary coil on the Tx side, where current is induced through coupling from the primary coil. Since the Tx coil consists of two coils, transmission to the Rx occurs through the coil with the higher coupling coefficient, determined by the misalignment state. Additionally, by controlling this current using a varactor placed on the auxiliary coil, an optimal magnetic flux is generated based on the alignment condition and/or the size of the Rx coil. In perfect alignment, the auxiliary coil focuses the flux from the Tx to the Rx coil, maximizing transfer efficiency. In misalignment scenarios, the current on the auxiliary coil is adjusted to shift the effective center of the Tx coil, achieving the strongest alignment of the magnetic flux traversing the Rx coil. This adjustment, which can be controlled adaptively based not only on the degree of misalignment but also on the size of the Rx coil, enables virtually null-free operation across varying misalignment conditions and for different Rx sizes. Furthermore, as this multifunctionality of the proposed system is achieved with a minimal number of additional components-just a single auxiliary coil and a single varactor-the impact on the overall quality factor (*Q*) of the system is minimized, contributing to the higher efficiency. In a size-symmetric system, where the Tx and Rx coils have the same size, the efficiency reaches 98.1% in perfect alignment and remains above 60% with up to 135% misalignment relative to the largest coil dimension. In a size-asymmetric system, with the Rx coil reduced to a quarter of the Tx coil, the efficiency is 96.1% in perfect alignment and remains above 60% up to 95% misalignment. Despite its enhanced practicality through a simple structure featuring only one auxiliary coil and an asymmetric configuration integrated solely on the Tx side, the proposed technique surpasses previous methods by delivering significantly superior performance. Moreover, it demonstrates unprecedented tolerance to both misalignment and smaller Rx coil sizes, which is frequently encountered in practical applications.

## Introduction

Wireless charging has become increasingly prevalent and has now matured to the point where its speed is comparable to that of wired charging. Wireless power transfer (WPT), which is one of the core technologies of wireless charging, is being developed in various ways, and research is actively being conducted to improve its performance and practicality. Inductive coupling, based on which the near-field WPT systems generally operate on, is so effective that the transfer efficiency is very high when the transmitting (Tx) and receiving (Rx) coils are aligned well. However, as the misalignment increases, the coupling coefficient reduces, and therefore the transfer efficiency. Although the decrease is monotonic and rather slow for low misalignment levels, it becomes rapid as the misalignment increases. At a specific point, the net magnetic flux traversing the Rx coil vanishes, leading to a transmission dead point with no further transmission occurring.

To overcome the problem of misalignment, various methods have been employed. These methods include using sensor coils to detect misalignment^1^, using metamaterials to enhance efficiency^2–4^, using multiple coil loops^5,6^, positioning Rx coils at vertical angles^7^, and using a stack of multiple coils^8^. In many cases, to maintain a consistent transfer efficiency regardless of the misalignment between the Tx and Rx, the coil array is widely spread out on the transmitting end so that the receiver is always positioned on top of the Tx coil^9–12^. In baseball terms, this is akin to having multiple catchers to catch wild pitches thrown by the pitcher. The need for a significantly larger Tx coil than the Rx coil and the resulting performance deterioration at equal coil sizes renders these techniques less practical.

Among previous techniques, the reconfiguration coil^13^, relies on an array of assistant coils loaded with varactors. When the Tx and Rx coils are misaligned, the electrical size and/or shape of the coil can be reconfigured to achieve the effect of improved alignment. This compensates for reduced transfer efficiency due to misalignment, maintaining high efficiency even when the misalignment is significant. One of the great advantages of this technique is that it can be applied to any existing coils, regardless of their shape, simply by integrating the array of assistant coils. However, the operation is rather complex as it requires all assistant coils to be loaded with varactors, which sometimes must be tuned independently. Moreover, the requirement of a number of independently tunable varactors to cover complete 2D misalignment not only increases the system’s complexity but also potentially impacts efficiency due to the varactors’ *Q*. Most importantly, although the basic principle is different from other techniques that fundamentally rely on an increased number of Tx coils to tackle misalignment problems, this technique still suffers from degraded performance when the Rx coil is smaller than the Tx, which is the case in virtually all practical situations. This is also common for the stacked coil^8^, which stacks the auxiliary coils of different sizes to construct a multi layer coil.

In this study, we present a novel multifunctional coil technique, representing a significant advancement in several aspects. Firstly, the proposed method simplifies the entire system by integrating just one smaller-sized auxiliary coil on the same layer as the primary coil, eliminating the need for additional volume and making the structure and operation much simpler than previous similar techniques. Furthermore, this approach minimizes the reduction in *Q* factor as only one varactor is required. The reduction in the quality factor (*Q*) resulting from an increased number of varactors can have a detrimental effect on the overall performance. However, the proposed technique mitigates this impact by adopting an asymmetric system configuration with a minimum number of varactors. This design choice helps to minimize the adverse effects on system performance. In contrast, previous technologies, such as the reconfigurable coil^13^ and stacked coil^8^, rely on multiple assistant coils positioned on a separate layer(s) from the primary coil. As a result, these approaches not only lead to an increase in the overall volume but also impose significantly more complex structural and operational requirements. Most importantly, the outstanding performance of our technique remains consistent even in size-asymmetric systems where the Rx coil is smaller than the Tx coil, regardless of the alignment condition. The varactor on the auxiliary coil optimizes the current on this coil to focus the flux from the transmitter (Tx) to the receiver (Rx) when they are well aligned. In contrast to the conventional 3- or 4- coil system, where the resonant frequency of the resonant coil(s) is fixed^14^, the proposed technique involves tuning the resonant frequency of the auxiliary coil depending on the misalignment. This enables the generation of an optimal magnetic field in both well-aligned and misaligned states. The integration of these multiple functions into a single auxiliary coil highlights the practical superiority of our technique compared to previous approaches.

Experimental results for a size-symmetric system consisting of rectangular Tx and Rx coils with dimensions of 100 $\times$ 100 mm$^2$ demonstrate that the efficiency reaches 98.1% in the perfectly aligned state. Remarkably, the efficiency remains above 60% even with misalignments of up to 135% relative to the largest coil dimension. Similarly, in the size-asymmetric system, where the Rx coil is a quarter of the Tx coil, the efficiency achieves 96.1% in perfect alignment and remains above 60% with misalignments of up to 95%. Unlike existing systems that suffer from transmission dead spots, resulting in a complete failure of wireless power transfer, the proposed system eliminates these dead spots, enabling virtually null-free operation. Consequently, the efficiency of the proposed system closely follows the ideal misalignment graph, where the efficiency decrease is primarily attributed to reduced coupling between the coils, regardless of the coil size ratio. This characteristic represents a significant advancement compared to conventional designs, as it ensures reliable power transfer even under misaligned conditions, irrespective of the coil size.

## Fields in multifunctional coil system

**Figure 1:**
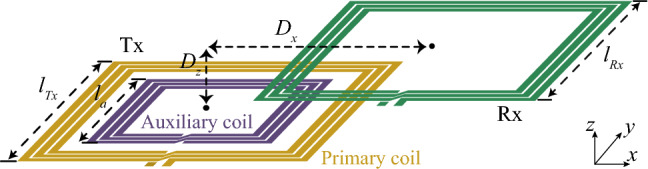
Proposed multi-functional coil system (size-symmetric).

Figure 1 depicts the proposed asymmetric configuration of the multifunctional coil system, where an auxiliary coil is integrated with the primary coil only on the Tx side, while the Rx coil remains conventional. The smaller size of the auxiliary coil allows for its integration on the same layer as the primary coil, eliminating the need for volume increase that was required by previous approaches based on coil arrays^8,13^. The analysis of the relationship between the input and output, as derived in Appendix A, suggests that the decrease in mutual inductance due to misalignment between Tx and Rx coils, and/or a reduction in Rx coil size can be compensated for by adjusting the resonant characteristics of the auxiliary coil, specifically the varactor capacitance that loads the auxiliary coil. Additionally, depending on the degree of misalignment between the Tx and Rx coils and their size ratios, the varactor capacitance can be tuned to optimize the current ratio between the primary and auxiliary coils on the Tx side to produce an optimal magnetic field that maximizes power transfer efficiency, regardless of the alignment condition and/or Rx size. Additionally, the symmetric design enables bidirectional misalignment compensation not only in the $\pm x$ but also in $\pm y$ directions.

### Magnetic flux in perfect alignment

**Figure 2:**
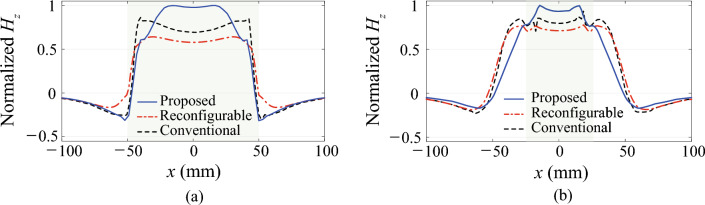
Normalized magnetic field ($H_z$) traversing Rx coil in perfectly-aligned state when size of Rx is: (**a**) 100 $\times$ 100 mm$^2$ and (**b**) 50 $\times$ 50 mm$^2$.

**Table 1:** Coil design parameters.

	Proposed		Reconfigurable coil		Conventional
	Primary coil	Auxiliary coil	Primary coil	Auxiliary coil	
$N*$	12	12	7	7	12
$l_{Tx}$	100 mm	–	100 mm	–	100 mm
$l_{a}$	–	70 mm	–	40 mm	–
$l_{Rx}$	100 mm and 50 mm		100 mm and 50 mm		100 mm and 50 mm
$\textit{w}$	0.5 mm	0.5 mm	0.5 mm	0.5 mm	0.5 mm
$\textit{g}$	0.2 mm	0.2 mm	0.2 mm	0.2 mm	0.2 mm
$\textit{h}$	0.8 mm	0.8 mm	0.8 mm	0.8 mm	0.8 mm
$\textit{t}$	70 μm	70 μm	70 μm	70 μm	70 μm
$\textit{L}$	31.14 μH	19.78 μH	13.92 μH	9.45 μH	31.14 μH

*Number of turns.

Figure 2 presents the magnetic field distribution at 6.78 MHz for the three (proposed, reconfigurable and conventional) systems, obtained using ANSYS HFSS^15^ when the transmitter (Tx) and receiver (Rx) coils are perfectly aligned ($D_x$ = 0). The design parameters of all three systems are summarized in Table 1 and Fig. 1, where *w* represents the width of the conductor, *g* represents the gap between the turns, *h* represents the thickness of the conductor, *t* is the conductor thickness, and *L* is the equivalent inductance. For all three systems assumed the excitation from the same source under perfect matching condition for 50 $\Omega$ source and load impedances. The magnetic field passing through the area of the Rx coil in the $\pm z$ direction is determined immediately below the Rx coil, along $y=0$. All magnetic fields shown in the figures are normalized with respect to the largest value, corresponding to that of the proposed system for both the size-symmetric and size-asymmetric cases. In Fig. 2a, the magnetic field generated is depicted when the Rx coil has a size of 100$\times$100 mm$^2$ ($l_{Rx}$ = 100 mm). Conversely, Fig. 2b displays the magnetic field generated when the Rx coil size is decreased to 50$\times$50 mm$^2$ ($l_{Rx}$ = 50 mm). Both cases assume a Tx coil with a size of 100$\times$100 mm$^2$. A separation distance of DZ=15 mm between the Tx and Rx coils is selected, aligning with the Qi A11 standard^16^. The smaller Rx coil serves as a model for wireless chargers designed to accommodate multiple devices. For the proposed system, the primary coil is integrated with an auxiliary coil size of 70$\times$70 mm$^2$. For comparison purposes, the magnetic fields for two other systems are included: the 2$\times$2 reconfigurable coil system, which extends the system demonstrated in a previous work^13^ to cover misalignment in both the $\pm x$ and $\pm y$ directions, and the conventional coil system without any additional coils. In the two systems with auxiliary coils, the varactors are tuned to maximize the transfer efficiency.

In Fig. 2a, it can be observed that the proposed system exhibits the highest intensity and most focused magnetic field traversing the Rx coil. While the conventional system has a slightly lower field intensity, it is distributed more uniformly along the Rx coil and becomes even stronger towards the edges. However, the focusing effect of the proposed system outperforms, resulting in an estimated transfer efficiency of 98.1%, calculated using Ansoft HFSS, which is slightly higher than the 97.8% achieved by the conventional system. On the other hand, the reconfigurable coil demonstrates the weakest field intensity due to the relatively lower *Q* of smaller auxiliary coils and varactors. Consequently, the expected transfer efficiency for the reconfigurable coil is 96.7%, which is the lowest among the three. The transfer efficiency employed in this work is the maximum power transfer efficiency, as demonstrated in Appendix B. This stands as a widely accepted metric for assessing the performance of coil systems for wireless power transfer applications^2,17–19^.

The effectiveness of the proposed system in focusing the magnetic field becomes more evident as the size of the Rx coil decreases, as shown in Fig. 2b. In this scenario, the proposed system excels by delivering the highest magnetic field strength to the Rx coil. In contrast, the conventional coil exhibits a weaker magnetic field that is spread over a larger area, extending beyond the Rx coil. As a result, the magnetic fields outside the Rx coil do not contribute to the transfer efficiency. Similarly, the reconfigurable coil system faces challenges due to the lower *Q* of the smaller auxiliary coils. This system relies on reconfiguring the electrical size and/or shape of the Tx coil, which presents difficulties in achieving enhanced efficiency for smaller Rx coils in the perfectly aligned state. The expected transfer efficiency of 96.2% for the proposed system serves as clear evidence of its effectiveness when compared to the efficiencies of 93.7% and 90.7% achieved by the conventional and reconfigurable coil systems, respectively.

**Table 2:** Optimal varactor capacitances for propopsed systems in Fig. 2.

Rx size (mm$^2$)	C (pF)
100 $\times$ 100	12.18
50 $\times$ 50	16.24

By tuning the varactor in the auxiliary coil, the proposed system can accommodate various sizes of Rx coils in the perfectly aligned state. In the size-symmetric system shown in Fig. 2a, the optimal varactor capacitance is relatively low, as shown in Table 2, resulting in weak current induced on the auxiliary coil. Consequently, the primary coil plays a more dominant role in maximizing the transfer efficiency to an Rx coil of the same size. In contrast, in the size-asymmetric system depicted in Fig. 2b, the varactor is adjusted to allow more current flow through the auxiliary coil. This adjustment enhances the dominance of the smaller-sized auxiliary coil, thereby maximizing the transfer efficiency to Rx coils with smaller sizes. The reconfigurability provided by the proposed system enables efficient power transfer across a range of Rx coil sizes.

### Magnetic fieds in misaligned state

**Figure 3:**
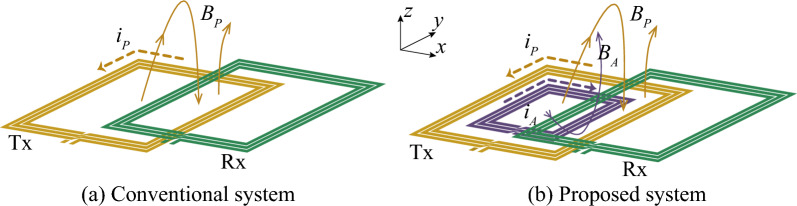
Illustration of magnetic fields when Tx and Rx are misaligned in *x* direction.

**Figure 4:**
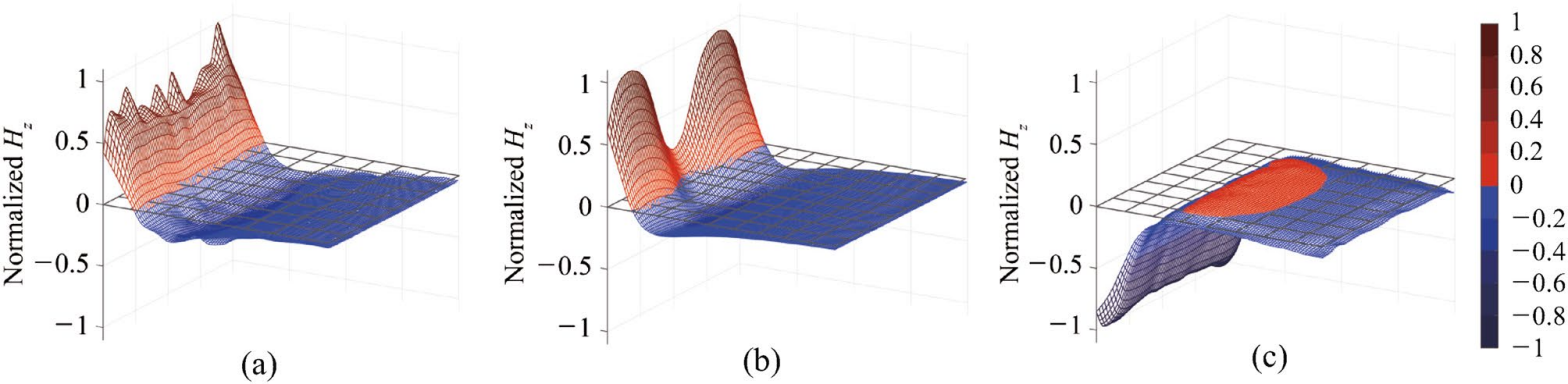
Normalized magnetic field ($H_z$) traversing Rx coil at transfer null (or local minimum) point of size-symmetric (**a**) Conventional (**b**) reconfigurable and (**c**) proposed systems.

Figure 3a illustrates the magnetic flux of the conventional coil system when the Rx coil is positioned at the transfer null point of the Tx. Subscripts *P* and *A* denote primary and auxiliary, respectively. At this specific point, the flux passing through the Rx coil in opposite directions becomes equal, resulting in a net zero flux. Consequently, no power transmission occurs because the Rx coil appears electromagnetically invisible to the Tx coil.

However, in the proposed system, the varactor is adjusted to ensure that the current on the auxiliary coil flows in the opposite direction to that of the primary coil. This adjustment is achieved when the varactor capacitance exceeds the value required to resonate the auxiliary coil at the operating frequency, which, in this study, is 6.78 MHz. As shown in Fig. 3b, this configuration allows the magnetic flux from both the auxiliary coil and the primary coil to traverse the Rx coil in the same direction. As a result, the proposed system achieves the strongest possible alignment of the magnetic flux passing through the Rx coil, effectively eliminating the transfer null point.

Figure 4 provides a comparison of the magnetic field in the ±z directions within the Rx coil, located 1 mm below it, for the three systems at their transfer null or local minimum points. The reference point at 0 mm is set with the end of the Rx coil in Fig. 3. The magnetic fields for all three systems in the size-symmetric configuration, excited by the same source under perfect-matching condition for 50 $\omega$ source and load impedances, are shown. Additionally, a 2D distribution is provided at the bottom to aid understanding of all six cases.

In the range of 0 $\le l_{Rx} \le$ 10 mm, the magnetic field generated by the conventional system exhibits relatively strong traversal through the Rx coil in the $+z$ direction. Although the field in the remaining range of $l_{Rx}$ is relatively weak, it traverses a significantly larger portion of the Rx coil in the opposite ($-z$) direction. As a result, the combined effects cancel each other out, leading to a transfer efficiency of 0%, which is calculated using a full-wave simulator Ansoft HFSS^15^. On the other hand, the reconfigurable coil system manipulates the magnetic field to achieve stronger alignment, particularly in the $-z$ direction in this scenario, resulting in a relatively high simulated transfer efficiency of 51.8%. However, the proposed system outperforms both systems by achieving significantly stronger alignment of the magnetic field predominantly in the $-z$ direction. As a result, the calculated transfer efficiency reaches as high as 75.2%, which is significantly superior to the other two systems.

**Figure 5:**
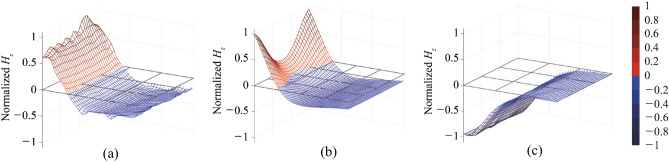
Normalized magnetic field ($H_z$) traversing Rx coil at transfer null (or local minimum) point of size-asymmetric (**a**) Conventional (**b**) reconfigurable and (**c**) proposed systems.

The superior ability of the proposed system to achieve stronger alignment of the magnetic field to compensate for the degraded efficiency due to misalignment is even more pronounced in the size-asymmetric system, particularly when the Rx coil size is smaller, such as 50 $\times$ 50 mm$^2$ as shown in Fig. 5. As the size of the Rx coil reduces, wireless power transfer systems generally experience lower transfer efficiency due to reduced coupling. In the case of the conventional system, as shown in Fig. 5a, the magnetic field traverses the Rx coil in opposite directions, resulting in a transfer efficiency of 0%.

Furthermore, the reduced size of the Rx coil in the size-asymmetric system undermines the ability of the reconfigurable coil system to align the magnetic field effectively. Similar to the conventional coil system, the magnetic field generated by the reconfigurable coil system traverses the Rx coil in both directions, leading to significant cancellation and resulting in a transfer efficiency of only 9.0%. As mentioned earlier, the reconfigurable coil system relies on adjusting the size and/or shape of the Tx coil using the 2 $\times$ 2 coil array. While this approach can compensate for misalignment between the Tx and Rx coils, it encounters challenges when the misalignment is such that the projection of the Rx coil on the Tx coil is smaller than the assistant coil(s).

In contrast, the proposed system effectively compensates for the degraded efficiency due to misalignment, even for a smaller Rx coil size. This compensation is achieved by controlling the ratio of current in the auxiliary coil and the primary coil, which flow in opposite directions. The shift of the effective center improves the effective alignment, compensating for the transfer efficiency degradation caused by misalignment. For instance, increasing the varactor capacitance enhances the current in the auxiliary coil, resulting in a shift of the effective center of the Tx coil in the $+x$ direction in Fig. 3b, towards the Rx. Thus, a superior tolerance to the size of Rx coils is achieved in compensating the lateral misalignment in WPT systems. Comparison of the magnetic field in Fig. 5c provides clear evidence of the superior capability of the proposed system to align magnetic fields. In this case, all magnetic flux is in the same direction across the entire Rx, resulting in a simulated transfer efficiency of 62.2%, which is significantly higher compared to the other two systems. This exceptional performance highlights the superiority of the proposed system in achieving consistent and reliable performance, not only in terms of misalignment but also regardless of the size of the Rx coil.

**Table 3:** Optimal varactor capacitances at local minimum.

Rx size	Proposed	Reconfigurable coil			
(mm$^2$)	C (pF)	C$_1$ (pF)	C$_2$ (pF)	C$_3$ (pF)	C$_4$ (pF)
100 $\times$ 100	35.18	110.05	10.69	10.69	110.05
50 $\times$ 50	28.75	110.05	10.69	10.69	110.05

Table 3 provides the optimal varactor capacitances for the proposed coil system at the local minimum point of transfer efficiency. As mentioned earlier, the varactor capacitance determines the effective center of the Tx coil, which represents the point where the alignment of the fields from the primary and auxiliary coils is the strongest. In Fig. 3, the local minimum point is observed at $D_x$ = 80 mm for the size-symmetric system, indicating that the effective center should be located around this position. Therefore, a higher varactor capacitance is necessary to induce a stronger current in the auxiliary coil, allowing the effective center to be shifted further away in the $+x$ direction. In contrast, the local minimum point occurs at $D_x=$ 58 mm for the size-asymmetric system, suggesting that the effective center should be shifted less than the size-symmetric system in the $+x$ direction. The lower varactor capacitance required in this case results in a weaker current in the auxiliary coil. As a result, the primary coil takes on a more dominant role, enabling the effective center to be shifted less in the $+x$ direction.

Table 3 also presents the required varactor capacitances for the reconfigurable coil system at its local minimum point of transfer efficiency, revealing a critical limitation of this system. The fact that the varactor capacitances are the same for both the size-symmetric and size-asymmetric systems indicates that the optimal fields are achieved only for the size-symmetric configuration. This implies that the reconfigurable coil system has limited tolerance to the size of the Rx coil, especially when it is smaller. This limitation is further supported by the significantly low transfer efficiency of 9.0%, emphasizing the system’s struggle to effectively compensate for misalignment for smaller Rx coil sizes, which is commonly encountered in practical applications.

## Experimental verification

For the purpose of demonstration, the proposed coil system was designed with the assumption that the size of the primary coil in the transmitter is 100 $\times$ 100 mm$^2$ with 12 turns. The receiver (Rx) coil size is also assumed to be the same, i.e., 100 $\times$ 100 mm$^2$ with 12 turns. The conductor width of all turns is *w* = 0.5 mm. The critical aspect is the design of the auxiliary coil to achieve the strongest alignment of the magnetic flux across the Rx coil in various misalignment states. Firstly, its size must be determined. In general, a larger auxiliary coil offers better performance in both aligned and misaligned states. However, it should not be too large to overlap with the driving coil, which is on the same layer. In this work, a size of 70 $\times$ 70 mm$^2$ is chosen. Secondly, parameters such as the number of turns (*N*) and the gap (*g*) between the turns are optimized. While the gap has a minor effect on the overall performance, the selection of N for the auxiliary coil must be made carefully, as there is a trade-off between the total flux and quality factor (*Q*) due to increased ohmic loss. In this work, *N* = 12 is chosen. Finally, the required range of varactor capacitance is determined by post-processing the full-wave simulated transmission, yielding the highest transfer efficiency. The final design parameters are summarized in Table 1.

To ensure a comprehensive performance comparison, two additional coil systems were fabricated: the reconfigurable coil system^13^ featuring a 2 $\times$ 2 array of assistant coils, and a conventional coil system without auxiliary coils. The number of turns for the assistant coils is determined to maximize the magnetic flux while minimizing the impact on the quality factor (*Q*). To maintain fairness in the comparison, these two coil systems were designed to match the size specifications of the proposed system. The receiver (Rx) coils, designed with two different sizes, 100 $\times$ 100 mm$^2$ and 50 $\times$ 50 mm$^2$, are shared by all three systems. The final design parameters are summarized in Table 1.

**Figure 6:**
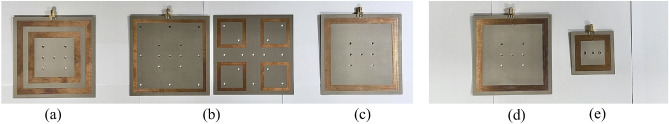
Photograph of the meaurement setup and fabricated coils (**a**) Proposed (Tx) (**b**) Reconfigurable (Tx, left: driving, right: assistant coil array) (**c**) Conventional (Tx) (**d**) Rx (100 $\times$ 100 mm$^2$) (**e**) Rx (50 $\times$ 50 mm$^2$).

**Figure 7:**
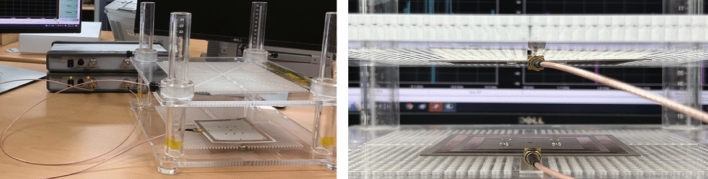
Photograph of the meaurement setup.

All three coils, namely the proposed, reconfigurable, and conventional coils, are fabricated on a Taconic RF-30 substrate with a relative permittivity ($\epsilon _r$) of 3. The substrate has a thickness of 0.8 mm and dimensions of 110 $\times$ 110 mm$^2$. The copper coils themselves have a thickness of 70 mm. The optimal varactor capacitances for achieving maximum efficiency are determined through post-processing of the full-wave simulated results in a circuit simulator. The required capacitance range for the proposed system falls between 12.18 pF and 94 pF, which is achieved using a SMV1702-011LF varactor diode from SKYWORKS in this work. Figure 6 displays a photograph featuring all the fabricated coils. The reconfigurable coil system adopts a two-layer configuration, necessitating an additional layer for the assistant coil array. Conversely, the proposed multifunctional coil system integrates the auxiliary coil within the same layer. Consequently, it is a single-layer system that avoids an increase in overall volume.

To evaluate performance under more practical conditions, the two systems that require additional coils are tested in an asymmetric configuration without the auxiliary coil or assistant coil array on the receiver (Rx) side. This approach acknowledges that integrating an additional coil layer may not be feasible in practical scenarios, especially due to the increase in volume, which is particularly relevant for mobile applications.

**Figure 8:**
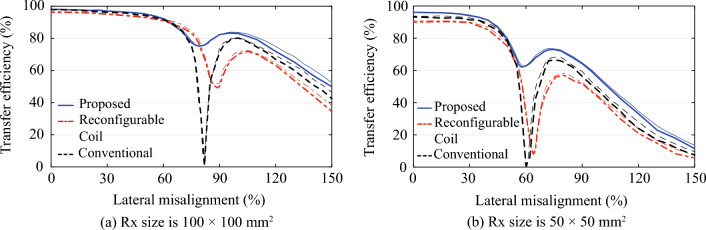
Transfer efficiency vs misalignment (thick: measured, thin: full-wave simulated).

Measurements were conducted using a generic acrylic fixture that provided precise control over the misalignment while maintaining a consistent distance of ($D_z$) = 15 mm between the Tx and Rx coils as shown in Fig. 7. The two-port transmission between the Tx and Rx coils was measured at a frequency of 6.78 MHz using an Anritsu MS46122B vector network analyzer (VNA) that was calibrated using the SOLT calibration kit TOSLKF50A-40. The bias for the varactors were supplied using a USB-3105 voltage output device. Measurements were taken at 5 mm intervals for a misalignment range ($D_x$) from 0 mm to 150 mm. When the efficiency changed rapidly, such as around the transfer nulls or local minimum points, measurements were taken at 1 mm intervals. In each measurement, the initially predetermined varactor capacitances were further fine-tuned to maximize the efficiency. The measured transmission was then post-processed using Keysight Advanced Design System^20^ to calculate the transfer efficiency^8,13^, which represents the maximum power transfer efficiency^2,17–19^. In practice, achieving this can be done by employing various adaptive matching networks^21,22^. The final results are shown in Fig. 8. The measurement is repeated for input power levels of −20 and +5 dBm. Because the results are virtually the same due to linearity, the responses for an input power level of −20 dBm are presented only. While not depicted in this context, the measured power factor was 1 regardless of the input power level. This indicates that in the proposed system, all the supplied power is effectively utilized as the active power. In order to provide a comprehensive comparison, the full-wave simulated results obtained from Ansoft HFSS are also included, and they exhibit excellent agreement with the measured data.

In the case of the size-symmetric system, shown in Fig. 8a, the proposed system demonstrates a high efficiency of 98.1% when perfectly aligned ($D_x$ = 0 %), surpassing the efficiencies of the other two systems, as anticipated. The conventional and reconfigurable coil systems achieved transfer efficiencies of 97.8% and 96.2%, respectively. It is important to note that the proposed system includes an auxiliary coil loaded with a varactor, which are not present in the conventional system. The inclusion of these components has the potential to reduce the overall quality factor (*Q*) of the system, leading to a lower efficiency than the conventional coil. Nevertheless, the higher efficiency achieved by the proposed system serves as clear evidence of its effective focusing performance as discussed previously. The higher efficiency of the proposed system, compared to the reconfigurable system, is partly due to the 1.17 times higher coupling coefficient (*k*) due to the larger number of turns (*N*). While a larger *N* generally implies more loss, the quality factor (*Q*), estimated from the measured impedance parameters, is still 1.80 times higher due to the reduced number of additional components.

The effectiveness of the proposed system becomes more evident with increasing misalignment. Up to a misalignment of 60% relative to the largest coil dimension, all three systems exhibit similar transfer efficiencies. However, beyond this point, a substantial difference is observed. The conventional system experiences a rapid decrease in transfer efficiency and encounters a transfer null at $D_x$ = 82%. Although the reconfigurable coil successfully eliminates the null, it still suffers from a significant decrease in transfer efficiency, reaching a local minimum of 49.2%. In contrast, the proposed system shows minimal change as misalignment increases. A significantly higher transfer efficiency of 75.1% is demonstrated at the local minimum point, which is also attributed to the 1.36 times higher coupling coefficient (*k*) and an estimated quality factor (*Q*) that is 2.16 times higher than that of the reconfigurable system. The transfer efficiency is not only higher than the other two systems in the entire measured misalignment range, but also sustains above 60% even with misalignments of up to 135%. This is 1.38 and 1.43 times higher than the reconfigurable coil system and conventional system, respectively.

The superior performance of the proposed multifunctional coil system becomes more evident in the size-asymmetric system, whose results are shown in Fig. 8b. The difference in the efficiency in the perfectly aligned state ($D_x$=0 %) became larger than the size-symmetric system. While the conventional and reconfigurable coil both suffered decrease in the efficiency due to the lower coupling associated with the reduced coil size, the proposed technique focuses the field adaptively to the smaller Rx coil and successfully minimize the decrease. The transfer efficiency is 96.1%, which is higher than 93.2% and 90.2% of the conventional and reconfigurable coil system. The remarkable ability of the proposed system to maintain efficiency even when the size of the Rx is reduced to a quarter is primarily attributed to the auxiliary coil. In the case of a size-symmetric system, the transmission is predominantly influenced by the primary coils of both the Tx and Rx in both the proposed and reconfigurable systems. In a size-asymmetric system, a decrease in the coupling coefficient (*k*) is unavoidable due to the smaller Rx coil size. Conversely, in the proposed system, the auxiliary coil within the Tx coil assumes the primary responsibility for transmission to the Rx. As a result, while the reconfigurable system undergoes a 2.64-fold reduction in the coupling coefficient, the proposed system effectively mitigates the reduction to 1.70 times. The 1.69 times higher quality factor also contributes to the proposed system’s ability to minimize the sacrifice in efficiency even in size-asymmetric conditions under perfectly aligned conditions.

Similar to the size-symmetric system, the transfer efficiency of the conventional system rapidly decreases and reaches a transfer null point at 60% misalignment. Surprisingly, the reconfigurable coil, which successfully eliminated the null in the size-symmetric system and achieved an efficiency close to 50%, encounters a virtual transfer null point where the efficiency drops as low as 7.9%. This can be attributed in part to the asymmetric configuration of the reconfigurable coil, where the assistant coil array is integrated only on the Tx side, rather than on both the Tx and Rx sides. However, the primary reason for this significant drop in efficiency is the limited freedom in adjusting the effective center of the reconfigured coil. As previously discussed, the varactor capacitances for the reconfigurable coil system are identical for both the size-symmetric and size-asymmetric systems, indicating that the magnetic field cannot be adaptively optimized based on the Rx coil size. Consequently, the effectiveness of the reconfigurable coil system is limited, especially for small Rx coils.

In contrast, the proposed system successfully eliminates the transfer null point and achieves a remarkably high transfer efficiency of 62.2% at the local minimum point. Once again, the efficiency of the proposed system surpasses that of the other two systems across the entire measured misalignment range. Compared to the reconfigurable coil system, the proposed system features a coupling coefficient that is 1.75 times higher and a quality factor that is 2.67 times higher. More importantly, the proposed system excels in optimizing the alignment of the magnetic flux passing through the Rx. While the former experiences a virtual null with an efficiency of only 7.9% at the valley, the latter maintains an efficiency above 60% even in the presence of a 95% misalignment. This represents a performance improvement of more than 1.41 times compared to the other two systems. While the efficacy of the proposed method diminishes as the vertical distance increases, it continues to outperform other systems.

Table 4 provides a comprehensive summary and comparison of the key performance metrics for all three systems. It is evident that the proposed system outperforms the other systems to provide superior tolerance to not only misalignment, but also Rx coil size. The multifunctional coil technique employed in the proposed system not only achieves the highest efficiency ($\eta$) when perfectly aligned by focusing the magnetic flux towards the Rx coil but also demonstrates effective misalignment compensation. The proposed system achieves the strongest possible alignment of the flux from the primary coil and the auxiliary coil, resulting in superior compensation for misalignment compared to the other systems. Additionally, the proposed system exhibits minimal performance degradation due to smaller Rx coil sizes. For the reconfigurable coil system, below 90% misalignment ($D_x\le 90\%$), the ratio of minimum to maximum efficiency is 0.51 for the size-symmetric system. This ratio reduces to 0.08, virtually 0, for the size-asymmetric system, highlighting the impact of reduced Rx size on the performance. It is worth noting that performance degradation due to smaller Rx sizes is a common phenomenon generally observed in WPT systems. However, the proposed multifunctional coil technique demonstrates superior performance. For the size-symmetric system, the ratio is 0.77, significantly higher than the reconfigurable system. More importantly, the ratio is maintained at 0.64, evidencing the proposed technique’s exceptional ability to minimize performance degradation caused by reduced Rx size. These results confirm the remarkable capability of the proposed multifunctional coil technique in consistently maintaining efficient performance, irrespective of misalignment and Rx coil size.

**Table 4:** Summary of measured results.

	Proposed system		Reconfigurable coil system		Conventional system	
Coil size ratio*	1	0.25	1	0.25	1	0.25
$\eta$ @ 0 mm	98.1%	96.1%	96.2%	90.2%	97.8%	93.2%
$\eta$ @ valley	75.1%	62.2%	49.2%	7.9%	0.0%	0.0%
($D_x$)	(80.0%)	(58.0%)	(89.0%)	(64.0%)	(82.0%)	(60.0%)
Max. $D_{x}$ @ $\eta \ge 60 \%$	135%	95%	83.9%	56.0%	77.5%	55.3%
Ratio of Min. $\eta$ to Max. $\eta$ @ $D_x \le$ 90%	0.77	0.64	0.51	0.08	0	0

*Ratio between sizes of Rx and Tx coils.

**Table 5:** Comparison with previous works.

	^13^	^23^	^24^	^12^	Proposed	
Tx (mm$^2$)	60 $\times$ 60	350 $\times$ 350	65 $\times$ 65	80 $\times$ 80	100 $\times$ 100	
Rx (mm$^2$)	60 $\times$ 60	350 $\times$ 350	22 $\times$ 22	40 $\times$ 40	100 $\times$ 100	50 $\times$ 50
Coil size ratio*	1	1	0.11	0.25	1	0.25
Frequency	6.78 MHz	3 MHz	145 kHz	15.3 MHz	6.78 MHz	
Configuration	Symmetric	Symmetric	Asymmetric	Asymmetric	Asymmetric	
System	Tunable assistant coils	Coil switching	Coil array	Coil array	Tunable auxiliary coil	
$D_z$	16.7%	57.14%	23%	18.75%	15%	
Max. $\eta$	96.0%	75.0%	98.0%	72.0%	98.1%	96.1%
Min. $\eta$ @ $D_x$ $\le$ 90%	46.3%	18.2%	30.0%	20.0%	75.1%	62.2%
(@$D_x$)	(@ 88.3%)	(@ 90.0%)	(@33.8%)	(@ 62.5%)	(@ 79.0%)	(@ 58.0%)
Ratio of Min. $\eta$ to Max. $\eta$ @ $D_x \le$ 90%	0.48	0.24	0.31	0.28	0.77	0.64
Max. $D_x$ @ $\eta \ge 60 \%$	85.0%	14.3%	27.7%	25.0%	135%	95%
$\eta$ @ $D_x'$** = 100%	71.0%	12.6%	32.0%	35.0%	83.0%	80.0%

*Ratio between sizes of Rx and Tx coils ** Relative to Rx coil size.

The recent efforts to enhance misalignment tolerance in inductively-coupled wireless power transfer (WPT) systems are summarized and compared in Table 5. Direct comparison is challenging due to variations in factors such as size, Tx/Rx distance, and frequency, all of which may significantly impact the transfer efficiency. Nonetheless, it is evident that the proposed system exhibits the highest tolerance to misalignment between the Tx and Rx coils, as well as variations in Rx coil sizes. Despite the disadvantage of being in an asymmetric configuration with the additional coil applied only on the Tx side, the size-symmetric system in this study outperforms previous size-symmetric and configuration-symmetric systems in all the aspects presented in the table. Furthermore, when compared to other size-asymmetric systems in the table, the size-asymmetric system in this work demonstrates unparalleled misalignment tolerance, maintaining 80% efficiency even at an extreme misalignment of 100% relative to the largest dimension of the smaller Rx coil. This clear verification highlights the multifunctional capability of the proposed technique, delivering unprecedented tolerance to misalignment and/or Rx coil size, thereby enabling virtually null-free operation.

## Conclusion

In conclusion, the multifunctional coil technique proposed in this paper has demonstrated remarkable efficacy in improving the transfer efficiency of inductively-coupled wireless power transfer systems, regardless of the alignment and/or the Rx size. Similar to previous techniques based on assistant coils, the proposed technique also relies on transitioning between coils with a more dominant coupling coefficient. However, it distinguishes itself by requiring a minimal number of additional components-just a single auxiliary coil and a single varactor. Consequently, the impact on the overall quality factor (*Q*) of the system is minimized, a critical factor in achieving higher efficiency regardless of the misalignment state. Further, by controlling the current on the varactor-loaded auxiliary coil, an optimal magnetic flux is generated. In perfect alignment, the current on the auxiliary coil is adjusted to focus the flux on the Rx coil, while in misalignment scenarios, it is tuned to shift the effective center of the Tx coil, achieving the strongest alignment of the magnetic flux passing through the Rx coil. The unique symmetric structure that co-centers the auxiliary coil with the primary coil provides greater flexibility in determining the location of this effective center compared to previous techniques, such as the reconfigurable coil system which is based on effectively-asymmetric 2$\times$2 auxiliary coils. Therefore, the proposed system achieves virtually null-free operation not only across varying misalignment conditions but also for different Rx sizes.

The exceptional performance has been verified with the experimental results. In a size-symmetric system where the Rx coil had the same 100 $\times$ 100 mm$^2$ size as the Tx coil, the efficiency achieved 98.1% in perfect alignment and remained above 60% even with misalignments of up to 135% relative to the largest coil dimension. In a size-asymmetric system, where the Rx coil size was reduced to a quarter of the Tx coil, the efficiency reached 96.1% in perfect alignment and remained above 60% up to 95% misalignment. Notably, the proposed technique exhibited an impressive minimum-to-maximum efficiency ratio below 100% misalignment, with a ratio as high as 0.77 for the size-symmetric system and a sustained ratio of 0.65 in the size-asymmetric system. These results clearly highlight the exceptional tolerance of the proposed technique to Rx coil size, ensuring outstanding performance even for small Rx coil sizes.

Moreover, the proposed technique offers the advantage of a very simple structure and operation, utilizing a single auxiliary coil in an asymmetric configuration on the transmitter (Tx) side. Nevertheless, the technique outperformed other methods that employed configuration-symmetric designs. The multifunctional nature of the technique showcased exceptional tolerance to variations in receiver (Rx) coil size, resulting in minimal degradation even with reduced Rx sizes. These features underscore the practicality and viability of the proposed technique for real-world applications.

### Supplementary Information

Supplementary Information.

## Data Availability

The datasets used and/or analysed during the current study available from the corresponding author on reasonable request.
