# Supplementary material for: Multifunctional coil technique for alignment-agnostic and Rx coil size-insensitive efficiency enhancement for wireless power transfer applications
Source: Sci Rep. 2023 Dec 21;13:22838. doi: 10.1038/s41598-023-50094-4 (PMC10739718; doi:10.1038/s41598-023-50094-4)
Supplement: Supplementary file 1 — Supplementary Information. [file 41598_2023_50094_MOESM1_ESM.pdf]

Appendix A

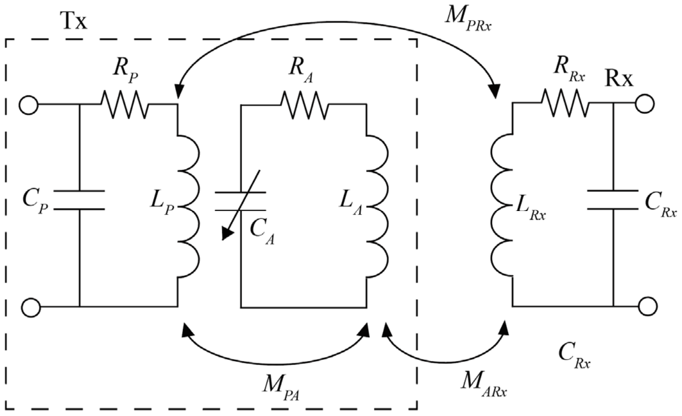

Supplementary figure 1. Equivalent circuit of the proposed system.

	Simulated			Measured		
	Inductance	Self-resistance	Self-capacitance	Inductance	Self-resistance	Self-capacitance
Tx (Primary coil)	31.14 µH	10.75 Ω	6.91 pF	32.09 µH	10.04 Ω	6.98 pF
Tx (Auxiliary coil)	19.78 µH	4.89 Ω	4.74 pF	-	-	-
Rx (100×100 cm^2)	31.14 µH	9.79 Ω	6.79 pF	32.09 µH	9.42 Ω	6.82 pF
Rx (50×50 cm^2)	11.3 µH	2.66 Ω	3.81 pF	11.6 µH	2.63 Ω	3.88 pF

Supplementary table 1. Inductance and Self-resistance and self-capacitance for the proposed coil system

	Rx: 100×100 cm^2	Rx: 50×50 cm^2
M_{PRx}	10.45 μH	1.11 μH
M_{ARx}	6.38 μH	1.43 μH
M_{PA}	9.72 μH	9.72 μH

Supplementary table 2. Calculated mutual inductances for the proposed coil system when Rx and Tx are perfectly aligned.

Supplementary figure 1 represents the equivalent circuit of the proposed system, where L denotes

inductance, and M signifies the mutual inductance between coils. Subscripts P and A refer to the primary and auxiliary in the Tx coil, while the subscript Rx designates the Rx coil. The varactor is modeled as a series connection of the capacitance C_A, which encompasses the self-capacitance of the auxiliary coil, and resistance R_A, which includes the self-resistance of the auxiliary coil. Since the self-capacitances C_P and C_{Rx} can be considered as parts of the matching networks, they are excluded from the analysis. Supplementary table 1 presents the self-resistances and self-capacitances and inductances of the individual coils employed within the proposed system. These values were determined through HFSS simulations and actual measurements. The mutual inductances for the proposed coil system, when the Rx and Tx are perfectly aligned, are summarized in Supplementary table 2. The impedance matrix of the Tx coil is

$$[Z] = \begin{bmatrix} Z_P & Z_{PA} \\ Z_{AP} & Z_A \end{bmatrix}, \quad (1)$$

where the subscripts denote primary and auxiliary, respectively. Therefore, when the voltage V_g is applied to the primary coil, the currents on the coils can be obtained using the linear equation below, which can be obtained using the Kirchoff's law:

$$\begin{bmatrix} V_S \\ 0 \end{bmatrix} = \begin{bmatrix} R_g + R_P + j\omega L_P & j\omega M_{PA} \\ j\omega M_{AP} & R_A + j\omega L_A + \dfrac{1}{j\omega C_A} \end{bmatrix} \begin{bmatrix} i_P \\ i_A \end{bmatrix}, \quad (2)$$

where R_g is the generator resistance, and i_P and i_A are the currents on the primary and auxiliary coils, respectively. From (2), the ratio between the two currents can be obtained as follows:

$$i_A/i_P = \frac{-j\omega M_{AP}}{R_A + j\omega L_A + \dfrac{1}{j\omega C_A}}. \quad (3)$$

From (3), it is evident that the current ratio between the auxiliary coil and the primary coil can be adjusted using the capacitance (C_A) of the loaded varactor of the auxiliary coil and the inductance L_A of the auxiliary coil.

Now, the relationship between the input and output of the two-port SISO (Single Input Single Output) WPT system is as follows:

$$\begin{bmatrix} V_S \\ 0 \\ 0 \end{bmatrix} = \begin{bmatrix} Z_P & j\omega M_{PA} & j\omega M_{PRx} \\ j\omega M_{PA} & Z_A & j\omega M_{ARx} \\ j\omega M_{PRx} & j\omega M_{ARx} & Z_{Rx} \end{bmatrix} \begin{bmatrix} i_P \\ i_A \\ i_{Rx} \end{bmatrix}, \quad (4)$$

where V_S is the source voltage and i_{Rx} is the current on the Rx coil. This leads to the following equations:

$$V_S = Z_P i_P + j\omega(M_{PA} i_A + M_{PRx} i_{Rx}), \quad (5)$$

$$0 = Z_A i_A + j\omega(M_{PA} i_S + M_{ARx} i_{Rx}), \qquad (6)$$

$$0 = Z_{Rx} i_{Rx} + j\omega(M_{PRx} i_P + M_{ARx} i_A). \qquad (7)$$

Multiplying (5) by $j\omega M_{PA}$, and (6) by Z_P results in the following expressions.

$$j\omega M_{PA} V_S = j\omega M_{PA} Z_P i_P - \omega^2 M_{PA}(M_{PA} i_A + M_{PRx} i_{Rx}), \qquad (8)$$

$$0 = Z_P Z_A i_A + j\omega M_{PA} Z_P i_P + j\omega M_{ARx} Z_P i_{Rx}. \qquad (9)$$

Also, multiplying (6) by $j\omega M_{PRx}$, and (8) by Z_P yields in the following expressions.

$$j\omega M_{PRx} V_S = j\omega M_{PRx} Z_P i_P - \omega^2 M_{PRx}(M_{PA} i_A + M_{SRx} i_{Rx}), \qquad (10)$$

$$0 = Z_P Z_{Rx} i_{Rx} + j\omega M_{PRx} Z_P i_P + j\omega M_{ARx} Z_P i_A. \qquad (11)$$

By substituting equation (8) into (9) and (10) into (11), the following expressions are obtained.

$$-j\omega M_{PA} V_S = Z_P Z_A i_A + j\omega M_{ARx} Z_P i_{Rx} + \omega^2 M_{PA}(M_{PA} i_A + M_{PRx} i_{Rx}), \qquad (12)$$

$$-j\omega M_{PRx} V_S = Z_P Z_{Rx} i_{Rx} + j\omega M_{ARx} Z_P i_A + \omega^2 M_{PRx}(M_{PA} i_A + M_{PRx} i_{Rx}). \qquad (13)$$

Equation (12) can be simplified as

$$i_A = \frac{-(j\omega M_{PA} V_S + i_{Rx} A)}{B}, (14)$$

where

$$A = j\omega M_{ARx} Z_P + \omega^2 M_{PA} M_{PRx},$$

$$B = Z_P Z_A + \omega^2 M_{PA}^2.$$

Similarly, (13) can be simplified as

$$-j\omega M_{PRx} V_S = i_{Rx} C + i_A A, \qquad (15)$$

where

$$C = Z_P Z_{Rx} + \omega^2 M_{PRx}^2.$$

Substituting equation (14) into (15) results in the following expression.

$$-j\omega M_{PRx} V_S = i_{Rx} C - \frac{(j\omega M_{PA} V_S + i_{Rx} A)}{B} \times A$$

$$= i_{Rx}\left(C - \frac{A^2}{B}\right) - \frac{A}{B}(j\omega M_{PA} V_S). \qquad (16)$$

By rearranging (17), the current on the Rx coil can be expressed as follows.

$$i_{Rx} = \left(C - \frac{A^2}{B}\right)^{-1} j\omega \left(\frac{A}{B} M_{PA} - M_{PRx}\right) V_S. \qquad (17)$$

This suggests that when the mutual inductance decreases between the Tx and Rx coils due to misalignment, it can be compensated for by adjusting the impedance of the auxiliary coil, specifically the varactor capacitance. Finally, equations (3) and (17) demonstrate that by tuning the varactor capacitance, we can control the current ratio between the primary and auxiliary coils on the Tx side. This ability allows us to electrically reconfigure the Tx coil, such as shifting the effective center of the coil to counteract reduced efficiency caused by misalignment. Consequently, efficiency can be maximized, irrespective of the alignment conditions.

Appendix B

The proposed coil system is two-port network for SISO-WPT (single input single output wireless power transfer) applications. Thus, the power transfer efficiency (PTE) denoted by η, which is the ratio of the output power to input power, can be formulated using *S*-parameters between the coils [1]:

$$\eta = \frac{P_{out}}{P_{in}} = \frac{(1 - |\Gamma_L|^2)|S_{21}|^2}{|1 - S_{22}\Gamma_L|^2 - |S_{11} - \Delta\Gamma_L|^2}, \quad (18)$$

where

$$\Delta = S_{11}S_{22} - S_{12}S_{21},$$

$$\Gamma_L = \frac{Z_L - Z_0}{Z_L + Z_0},$$

Z_0 is the reference impedance, which is 50 Ω in general, and Z_L is the load impedance. In this work, the efficiency is that in (18), under the perfectly matched conditions:

$$Z_L = Z_{in}^*,\ Z_S = Z_{out}^*.$$

In this case, the PTE becomes MPTE (maximum power transfer efficiency), which is expressed as follows [1].

$$\eta = \frac{1}{1 - \left|\Gamma_S^{\mathrm{opt}}\right|^2} |S_{21}|^2 \frac{1 - \left|\Gamma_L^{\mathrm{opt}}\right|^2}{\left|1 - S_{22}\Gamma_L^{\mathrm{opt}}\right|^2}, (19)$$

where

$$\Gamma_S^{\mathrm{opt}} = \frac{B_1 - \sqrt{B_1^2 - 4|C_1|^2}}{2C_1},$$

$$\Gamma_L^{\mathrm{opt}} = \frac{B_2 - \sqrt{B_2^2 - 4|C_2|^2}}{2C_2},$$

$$B_1 = 1 + |S_{11}|^2 - |S_{22}|^2 - |\Delta|^2,$$

$$B_2 = 1 + |S_{22}|^2 - |S_{11}|^2 - |\Delta|^2,$$

$$C_1 = S_{11} - \Delta S_{22}^*,$$

$$C_2 = S_{22} - \Delta S_{11}^*.$$

Thus, the coil-to-coil efficiency in (19) can be derived from the *S*-parameters between the coils. It is

also called the link efficiency [2]-[4], which serves as a widely adopted metric for quantitative evaluation of the performance of coils for WPT applications [5]-[7].

References

[1] Q. Yuan, "S-Parameters for Calculating the Maximum Efficiency of a MIMO-WPT System: Applicable to Near/Far Field Coupling, Capacitive/Magnetic Coupling," *IEEE Microwave Magazine*, 24(4), 40-48, 2023.

[2] T. S. Pham, T. D. Nguyen, B. S. Tung, B. X. Khuyen, T. T. Hoang, Q. M. Ngo, *et al*., "Optimal frequency for magnetic resonant wireless power transfer in conducting medium," *Scientific Reports*, 11(1), 18690, 2021.

[3] P. Darvish, S. Mekhilef, and H. A. B. Illias, "A novel S–S–LCLCC compensation for three-coil WPT to improve misalignment and energy efficiency stiffness of wireless charging system," *IEEE Transactions on Power Electronics*, 36(2), 1341-1355, 2020.

[4] M. J. Karimi, A. Schmid, and C. Dehollain, "Wireless power and data transmission for implanted devices via inductive links: A systematic review," *IEEE Sensors Journal*, 21(6), 7145-7161, 2021.y

[5] A. A. Shaier, A. A. Mohamed, H. Metwally, and S. I. Selem, "A new hollow solenoid receiver compatible with the global double-D transmitter for EV inductive charging," *Scientific Reports*, 13(1), 11925, 2023.

[6] D. Shan, H. Wang, K. Cao, and J. Zhang, "Wireless power transfer system with enhanced efficiency by using frequency reconfigurable metamaterial," *Scientific Reports*, *12*(1), 331, 2022.

[7] J. Zhang, J. Zhao, Y. Zhang, and F. Deng, "A wireless power transfer system with dual switch-controlled capacitors for efficiency optimization," *IEEE Transactions on Power Electronics*, 35(6), 6091-6101, 2019.

Appendix C

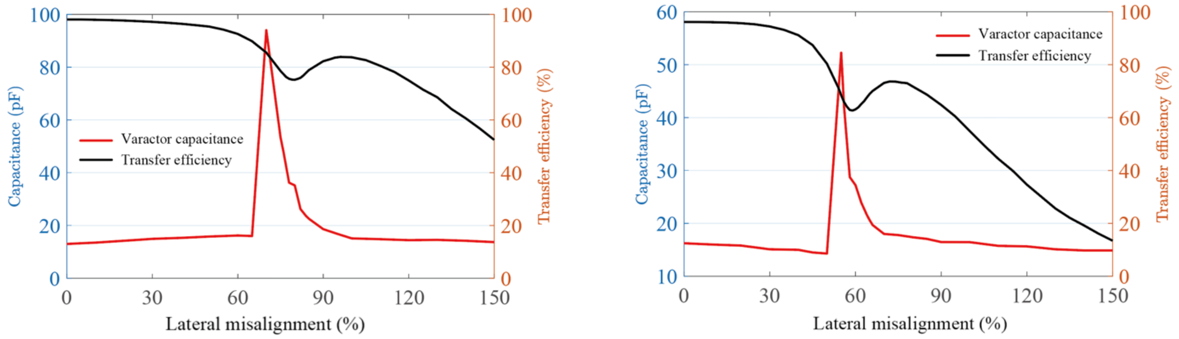

(a) 100×100 mm^2 (b) 50×50 mm^2

Supplementary figure 2. Required varactor capacitance and transfer efficiency versus lateral misalignment.

In Supplementary figure 2, the optimal varactor capacitances and the simulated transfer efficiency for lateral misalignment re provided.

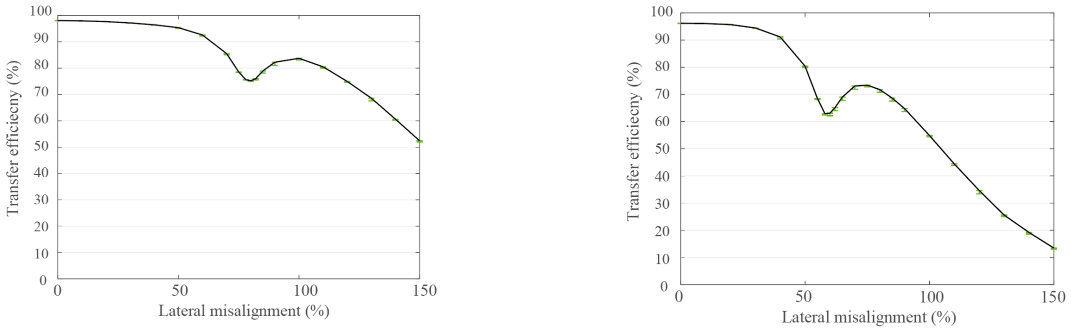

(a) 100×100 mm^2 (b) 50×50 mm^2

Supplementary figure 3. Simulated effect of ±5% capacitance error.

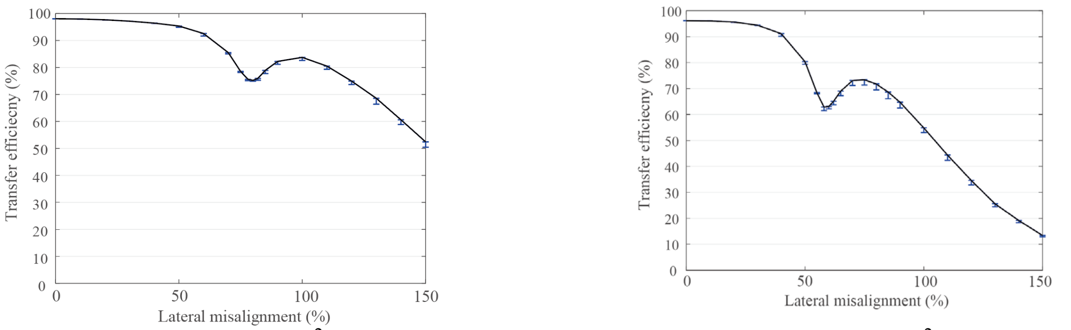

(a) 100×100 mm^2 (b) 50×50 mm^2

Supplementary figure 4. Simulated effect of ±10% capacitance error.

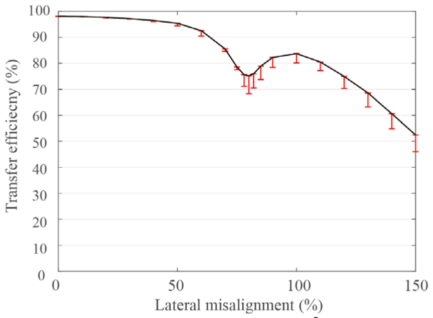

(a) 100×100 mm^2

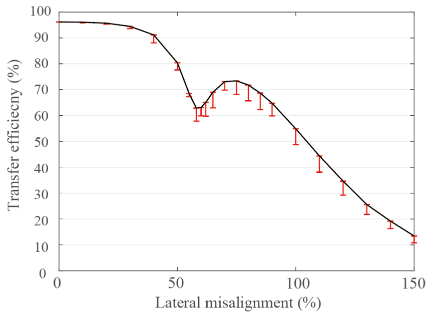

(b) 50×50 mm^2

Supplementary figure 5. Simulated effect of ±30% capacitance error.

Supplementary figures 3, 4, and 5 show the simulated effects of ±5%, ±10%, and ±30% errors in capacitance values. For comparison, the simulated efficiencies with the optimal capacitance is provided for all cases. Although a decline in efficiency becomes apparent when the capacitance error is heightened to ±30%, particularly in the vicinity and beyond the local minimum point, the efficiency demonstrates minimal variation between ±5% and ±10% errors, irrespective of the Rx size and/or alignment conditions. The system's resilience in maintaining efficiency within this margin highlights its robustness against variations in capacitance, offering consistent performance unless the deviation is substantially large.

Appendix D

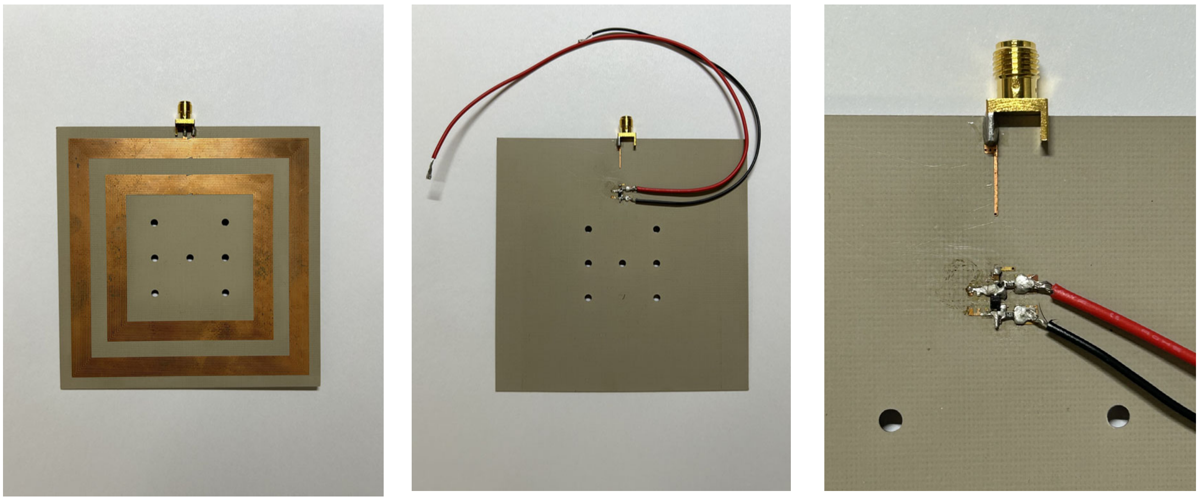

(a) Front side (b) Back side (c) Varactor

Supplementary figure 6. Photograph of the Tx coil.

Supplementary figure 6 presents photographs of the Tx coil, with the SMV1702-011LF varactor diode from SKYWORKS soldered on the back side of the substrate. Both ends of the varactor is connected to 100 kΩ resistors as RF choke. Additionally, a 10 nF capacitor is introduced as a DC block between the coil and the varactor.

Appendix E

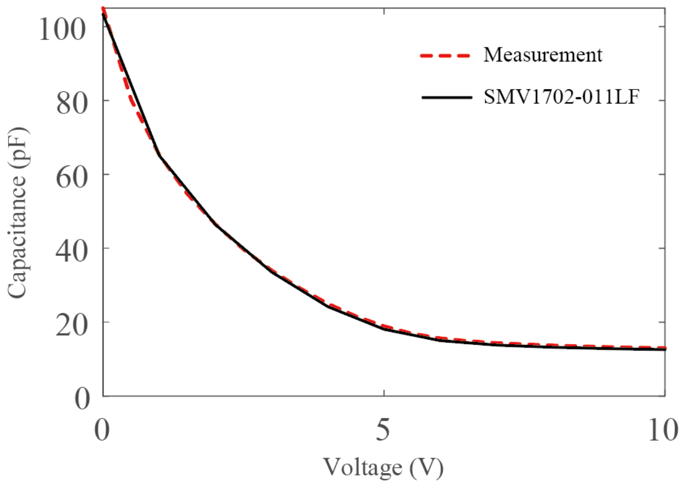

Supplementary figure 7. Measured capacitance vs voltage of varactor SMV1702-011LF.

Supplementary figure 7 illustrates the measured capacitance for the varactor SMV1702-011LF utilized in our experiments. The results demonstrate a strong agreement between our measured and those specified in the datasheet, affirming the reliability of our measurements.
